# A concern about plagiarism

**DOI:** 10.2349/biij.4.2.e22

**Published:** 2008-04-01

**Authors:** KH Ng, BJJ Abdullah, NA Kadri

**Affiliations:** 1 Department of Biomedical Imaging, Faculty of Medicine, University of Malaya, Kuala Lumpur, Malaysia; 2 Centre of Biomedical Engineering, University of Surrey, Guildford, Surrey, United Kingdom

The Biomedical Imaging and Intervention Journal (***biij***), like all other scientific journals, is concerned with the serious issue of plagiarism. The Publications Committee of the International Organization of Medical Physics (IOMP) has prepared an editorial on plagiarism, which is reproduced here with some modifications, with permission from the IOMP. In addition, this editorial is also consistent with the Policy on Plagiarism of World Association of Medical Editors (WAME) [1].

Plagiarism – from the Latin “plagiare,” meaning “to kidnap” – is defined as “the appropriation or imitation of the language, ideas and thoughts of another author and representation of them as one’s original work” [2]. Plagiarism is a serious breach of research ethics that, if committed intentionally, is considered research misconduct. Plagiarism may result in serious sanctions including public disclosure, loss of research funding, loss of professional stature, and termination of employment.

Plagiarism undermines the authenticity of research manuscripts and the journals in which they are published. It also compromises the integrity of the scientific process and public regard for science. Plagiarism violates the literary rights of individuals whose work has been plagiarised and the property rights of copyright holders. Violation of literary or property rights may result in legal action against the individual(s) committing plagiarism. Although plagiarism has existed since the beginning of science, it seems to be increasing because the World Wide Web facilitates finding and copying the work of others.

It is possible not only to plagiarise the work of others, but also one’s own work through reuse of identical or nearly identical portions of manuscripts without acknowledgment or citation. Simultaneous or subsequent submission of similar manuscripts with only minor differences and without citation between the manuscripts is, unfortunately, a rather common practice among authors hoping to acquire multiple publications from a research project. Scientific journals discourage this practice and usually will not permit it if exposed before publication. Occasionally, the same – or a very similar – article may be published in two journals because the journals reach different audiences and the article is of interest to both. This practice must be approved by the editors of both journals and the duplication must be acknowledged in each article.

When there is a possibility of plagiarism – often through an allegation of plagiarism by the original author, a reviewer or an interested third party – the journal’s editor should act quickly. The editor should examine the original material and the publication alleged to have performed plagiarism. If the editor concludes that no plagiarism has occurred, the accuser should be notified, and no further action is necessary.

If the evidence suggests that plagiarism may have occurred, the editor should contact the accused author(s), the author(s) whose work may have been plagiarised, and the copyright holder of the original material if different from the author(s). The correspondence should include the alleged plagiarising language and a copy of the original and suspected work. If all parties agree that plagiarism – whether intentional or unintentional – has occurred, a written letter of apology should be sent promptly by the offending author(s) to the editor and to the author(s) and copyright holder of the plagiarised work. If the offending work has been published, a notice of plagiarism, citing both the plagiarised and the offending articles and containing the exact text that has been plagiarised, should be published in the next available issue of the journal in which the offending article was published. The plagiarising author(s) must agree that all dissemination of the offending article will be accompanied by the notice of plagiarism.

If the accused author(s) denies that plagiarism has occurred, the editor must explore the accusation further, preferably through a mechanism already established by the journal to investigate allegations of scientific misconduct. All parties to the allegation should be encouraged to submit corroborating evidence, and the accused author(s) should be granted an opportunity – at no expense to the journal – to testify in person in defence against the allegation. The investigation should be concluded within a reasonable period of time (e.g., 3 months).

If the mechanism to investigate the allegation of plagiarism concludes in support of the allegation, then the process for the case in which plagiarism is admitted should be instituted. Further, the editor must decide whether the plagiarism should be reported to the guilty parties’ supervisor, employer, and/or professional organisation.

If the mechanism rules against the accusation of plagiarism, a letter stating this ruling should be provided to the accuser, the author(s) accused of plagiarism, the author(s) of the original work, and the copyright holder if different from the author(s). In either case, these actions should constitute closure of the allegation of plagiarism.

Self-policing is a major strength of the scientific community and suspected plagiarism should always be reported, even if the suspected plagiariser is a colleague or superior. An allegation of plagiarism is a serious accusation and should never be taken lightly. Further, laboratory directors, senior authors, and other individuals in leadership positions should educate junior members of a research team about responsible conduct in preparing scientific manuscripts for publication.

***biij*** fully supports the initiative in combating plagiarism and will try to closely adhere to the above detailed mechanism in handling reported cases of plagiarism. Although there are no such reports or accusations hitherto, the Editorial Board has taken the liberty of randomly checking the integrity of submitted papers for evidence of plagiarism. The Board has been using Google Scholar (http://google.com/scholar/) as a tool in the evaluation process, and is currently trying the services of the CrossCheck plagiarism detection service by CrossRef (http://crossref.org/).

***biij*** retains the right to alter the above mechanism in handling plagiarism cases should the currently worrying advent of plagiarism in scientific literature decrease in the future.
